# Obesity decreases excitability of putative ventral tegmental area GABAergic neurons

**DOI:** 10.1002/phy2.126

**Published:** 2013-10-23

**Authors:** Susumu Koyama, Mari Kawaharada, Hiroki Terai, Masahiro Ohkurano, Masayoshi Mori, Syohei Kanamaru, Shinichi Hirose

**Affiliations:** 1 Department of Psychosomatic Medicine, Faculty of Pharmaceutical Sciences, Fukuoka UniversityFukuoka, Japan; 2 Department of Pediatrics, School of Medicine, Fukuoka UniversityFukuoka, Japan; 3 Central Research Institute for the Pathomechanisms of Epilepsy, Fukuoka UniversityFukuoka, Japan

**Keywords:** Electrophysiology, fat intake, feeding behavior, neurons, obesity

## Abstract

Palatable food has reinforcing effects on feeding and accelerates obesity. Alteration of food-related behavior in obesity may promote maintenance of obesity. The ventral tegmental area (VTA) of the midbrain is important for food reward. However, it is unknown whether activity of VTA neurons is altered in diet-induced obesity. In this study, we examined VTA neuronal activity using an electrophysiological technique in diet-induced obese mice. Male 4-week-old mice were fed a high-fat diet or a standard diet for 5–6 weeks. Mice fed a high-fat diet gained greater body weight with heavier visceral fat compared with those fed a standard diet. Brain slice preparations were obtained from the lean and obese mice. Spontaneous activity of VTA neurons was recorded extracellularly. We found a negative correlation between firing frequency (FF) and action potential (AP) current duration in lean and obese mice VTA neurons. VTA neurons were classified as group-1 neurons (FF <5.0 Hz and AP current duration >1.2 msec) or group-2 neurons (FF ≧5.0 Hz and AP current duration ≦1.2 msec). FF, AP current duration, and firing regularity of VTA group-1 neurons were similar between lean and obese mice. Obese mice VTA group-2 neurons had a lower FF and shorter AP current duration compared with lean mice. In conclusion, obesity minimally affects VTA group-1 neurons, which are presumed to be dopaminergic, but decreases excitability of VTA group-2 neurons, which are presumed to be GABAergic. This differential effect may contribute to the pathophysiology of reward-related feeding in obesity.

## Introduction

Obesity is one of the most important public health issues in modern society. The increase in obesity is suspected to be associated with the increased prevalence of metabolic syndrome and type 2 diabetes mellitus. Glycemic impairment in children and adolescents progresses faster compared with adults, in that 25% of severely obese children with impaired glucose tolerance develop type 2 diabetes mellitus within 2 years (Weiss et al. 2005), while it takes 5–10 years in adults (Saad et al. 1988). Therefore, early life body weight control is critical to prevent obesity and a potential risk factor for rapidly progressing glycemic impairment.

Excessive intake of energy-rich foods is one of the most important factors contributing to the acceleration of obesity. Animal studies have shown that continuous consumption of high-fat diets makes rodents obese (Nishikawa et al. 2007; Bjursell et al. 2008; Donovan et al. 2009; Geiger et al. 2009; Li et al. 2009). Because palatable high-fat diets act as a natural reward, rodents continue consuming high-fat diets (la Fleur et al. 2007; Johnson and Kenny 2010). The hypothalamus contributes to the “homeostatic” aspect of feeding by regulating adequate energy intake, but fails to overcome the drive for reinforcing food stimuli, known as the “hedonic” aspect of feeding.

The mesolimbic dopamine system is important for the mediation of natural rewards such as foods. This reward circuit in the brain is mainly composed of the ventral tegmental area (VTA) and nucleus accumbens (NAcb). The VTA sends dopaminergic afferents to the NAcb. Increases in dopamine transmission in the NAcb are critical for reinforcing natural stimuli (Wise 2002; Robinson and Berridge 2003; Fulton 2010; Narayanan et al. 2010). The VTA is located in the ventromedial region of the mesencephalon (Franklin and Paxinos 2008). Neurons in the VTA are mainly classified into two neuronal groups (Grace and Onn 1989; Johnson and North 1992); dopamine neurons (Brodie and Dunwiddie 1987; Margolis et al. 2008) and γ-aminobutyric acid (GABA) neurons (Steffensen et al. 1998; Margolis et al. 2012). VTA dopamine neurons are the major neuronal subpopulation (∼70%), regulating dopaminergic tone in the NAcb (Di Chiara and Imperato 1988; Gonon 1988). VTA GABA neurons (∼30%) comprise several neuronal subtypes including inhibitory local circuit interneurons in the VTA (Van Zessen et al. 2012) and projection neurons to the NAcb (Van Bockstaele and Pickel 1995; Brown et al. 2012). In addition, glutamatergic neurons are present in the VTA (Yamaguchi et al. 2007; Nair-Roberts et al. 2008). Previous studies have shown that a small population of VTA neurons express vesicular glutamate transporter 2 (VGLUT2), and rarely co-express tyrosine-hydroxylase (TH), a marker for dopaminergic neurons, or glutamic acid decarboxylase (GAD), a marker for GABAergic neurons.

Ventral tegmental area dopamine and GABA neurons are also characterized by their electrophysiological properties. Both VTA dopamine and GABA neurons are spontaneously active and generate action potentials (APs) (Brodie and Dunwiddie 1987; Margolis et al. 2008, 2012). Thus far, it is generally considered that VTA dopamine neurons exhibit a slow firing frequency (FF) with broad AP duration, while VTA GABA neurons exhibit a fast FF with short AP duration (Brodie and Dunwiddie 1987; Grace and Onn 1989; Johnson and North 1992). However, these electrophysiological criteria are not always reliable for identifying dopamine and GABA neuronal populations in the VTA, because of considerable overlap of the properties of the two types of neurons (Lammel et al. 2008; Cohen et al. 2012; Margolis et al. 2012). Therefore, additional electrophysiological evidence is required to differentiate between the two VTA neuronal populations.

A recent study has shown that both VTA dopamine and GABA neurons regulate reward-related feeding behavior in mice (Van Zessen et al. 2012). We hypothesized that the neuronal activity of VTA neurons is altered in obesity induced by energy-rich high-fat diet intake. In this study, we used a diet-induced obese animal model comparable to obese children and adolescents, and examined the activity of two types of VTA neurons presumed to be putative dopamine and GABA neurons based on additional electrophysiological classification criteria.

## Methods

### Animals

Male 4-week-old imprinting control region (ICR) mice (Kyudo Co. Ltd., Saga, Japan) were housed in groups (*n* = 5 in a plastic cage; 30 × 25 × 18 cm) with a 12/12-h light-dark cycle schedule (lights on at 19:00). Mice were kept in a temperature- and humidity-controlled (20–24°C, 53–57%) room under specific pathogen-free conditions. Mice were given access to food and water ad libitum. Animals used in this study were treated in strict accordance with the U.S. National Institutes of Health Guide for the Care and Use of Laboratory Animals, and all experimental methods were approved by the Animal Care Committee of Fukuoka University.

### Diet intake

One group of mice aged 4 weeks was fed a standard diet (fat 13%, carbohydrate 60%, protein 27%, total energy 3.5 kcal/g) (CE-2: Clea Japan Inc., Tokyo, Japan) for 5–6 weeks. The other group of mice was fed a high-fat diet (fat 45%, carbohydrate 35%, protein 20%, total energy 4.7 kcal/g) (D12451: Research Diets, New Brunswick, NJ) for the same period. Food intake was measured at 10:00 am using a digital balance (TE15025, Sartorius Co., Guttingen, Germany).

### Body weight and visceral organs

Body weight was measured at 10:00 am on a digital balance (TE15025, Sartorius Co.). At 9–10 weeks, visceral organs including the liver, kidneys, and adipose tissues (mesenteric, perinephric, and epididymal) were weighed using the digital balance.

### Preparation of brain slices

Under anesthesia with pentobarbital (50 mg/kg), each mouse (9–10 weeks old) was killed and the brain quickly removed. The brain was placed in an ice-cold cutting solution consisting of (in mmol/L): 220 sucrose, 2.5 KCl, 2.4 CaCl_2_, 1.3 MgSO_4_, 1.24 NaH_2_PO_4_, 26 NaHCO_3_, and 11 d-glucose, which was constantly bubbled with 95% O_2_ and 5% CO_2_. A transverse brain slice, at a thickness of 400 μm, was cut using a vibrating blade brain slicer (7000 SMZ, Campden Instruments, Loughborough, U.K.). The brain slice was placed on a glass platform in a recording chamber (RC-22C, Warner Instruments, Hamden, CT) and perfused with an artificial cerebrospinal fluid (ACSF), consisting of (mmol/L): 126 NaCl, 2.5 KCl, 2.4 CaCl_2_, 1.3 MgSO_4_, 1.24 NaH_2_PO_4_, 26 NaHCO_3_, and 11 d-glucose, which was constantly bubbled with 95% O_2_ and 5% CO_2_. The ACSF was warmed to 35°C using an in-line solution heater, which was connected to a thermostatic temperature circulator (NTT-2200; Tokyo Rikakikai Co. Ltd., Tokyo, Japan). The temperature of the ACSF in the recording chamber was directly monitored by a digital thermometer (7001H; Netsuken, Tokyo, Japan). An S-shaped platinum frame was used to hold the brain slice in the recording chamber. The VTA, located between the interfascicular nucleus and the medial lemniscus in the horizontal axis, and between the paranigral nucleus and the red nucleus in the sagittal axis, was visually identified (Franklin and Paxinos 2008) under a binocular dissection microscope (M50, Leica, Germany).

### Electrophysiological recording

After approximately 1 h of perfusion with ACSF, blind extracellular recordings of the brain slice preparation were performed. Extracellular voltage-clamp recording at a holding potential of 0 mV is able to avoid disrupting the intracellular milieu, and provides a low impedance pathway through the patch for fast events occurring in a few milliseconds such as APs (Perkins 2006). Spontaneous AP currents were recorded using a Multiclamp-700B patch-clamp amplifier (Molecular Devices, Sunnyvale, CA). Microelectrodes were fabricated from glass capillaries (OD: 1.5 mm, ID: 0.86 mm) (BF150-86-10; Sutter Instrument Company, Novato, CA) on a P-97 puller (Sutter Instrument Company). The tip resistance of each electrode was 7–15 MΩ when filled with 0.9% NaCl. A depolarizing rectangular voltage pulse of 10 mV was applied to the electrode and negative pressure was gently applied to complete a loose patch. Seal resistance was usually less than 1 GΩ and periodically monitored during recordings. Membrane currents were filtered at 2 kHz and acquired at a sampling frequency of 10 kHz. Data acquisition was performed with a Digidata 1440A interface and pClamp software version 10.2 (Molecular Devices).

### Data analysis and statistics

Spontaneous firing was continuously recorded for 8.4 ± 0.8 min (*n* = 90) and an initial 120-sec long recording of spontaneous AP current generation was analyzed. AP currents were detected by their peaks of initial inward current component using a threshold-searching configuration in pClamp software (Molecular Devices). Duration between the peaks was estimated to be the interspike interval (ISI), and FF was also calculated. The coefficient of variation (CV) of the ISI was obtained by dividing the standard deviation of the ISIs by the mean ISI. AP current duration was measured between the initiation of the inward current component and subsequent outward current peak of the AP current, as previously described (Chieng et al. 2011; Margolis et al. 2012). Records including an AP current amplitude <10 pA and CV of ISI >1.0 were considered unstable and not used for analyses. ISI histograms were made as described by Cocatre-Zilgien and Delcomyn (1992). The number of bins was equal to the square root of the number of ISIs. Bin width was obtained by dividing the ISI range (maximum ISI – minimum ISI) by the number of bins. The normal distribution of the data was evaluated using the Kolmogorov-Smirnov test. When normality was confirmed, two-tailed Student's *t-*tests were used for comparisons between the two groups. To test for homogeneity of variance, the *F-*test was used. The relationship between FF and AP current duration of VTA neurons was analyzed using the Spearman rank correlation coefficient. Differences were considered statistically significant at *P* < 0.05. Numerical values are reported as mean ± standard error of mean (SEM). Graphing and statistics were conducted using Origin8 software (OriginLab, Northampton, MA).

## Results

### Lean and obese mice

**Figure 1:**
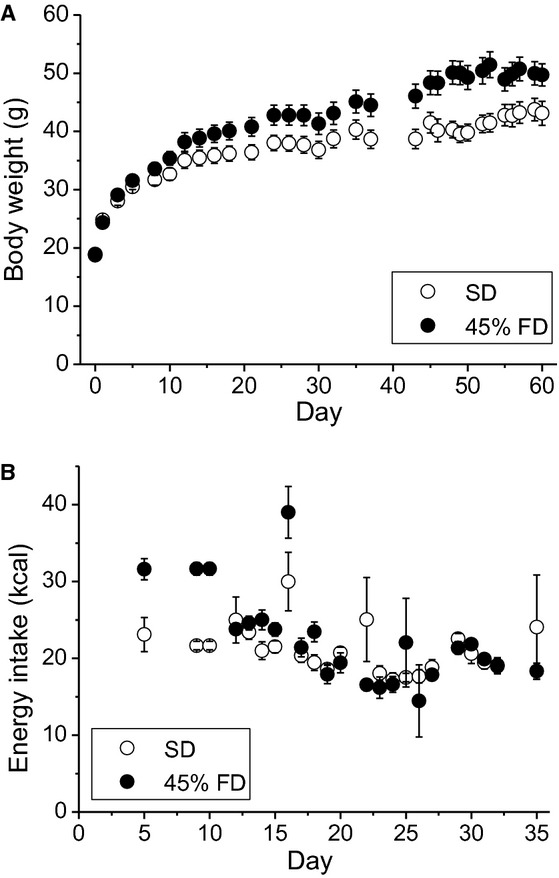
(A) Body weight increases of mice fed a standard diet (open circles, *n* = 9) and mice fed a high-fat (45%) diet (closed circles, n = 9). Body weight for the two groups was compared using two-way analysis of variance (ANOVA). (B) Daily energy intake of mice fed with a standard diet (open circles, *n* = 9) and mice fed with a high-fat diet (closed circles, *n* = 9). SD, standard diet; FD, fat diet. Values are ± SEM.

Figure 1A shows body weight increases in mice fed a standard diet and mice fed a high-fat (45%) diet. Mice fed with the high-fat diet gained greater body weight compared with mice fed with the standard diet (*P* < 0.0001), resulting in obese and lean mice. Figure 1B shows the daily energy intake for mice fed with the standard diet and the high-fat diet. Mice fed with the high-fat diet consumed greater energy from the diet in the initial periods of development compared with mice fed the standard diet. As shown in Table 1, we measured the visceral organ weight of lean and obese mice. Obese mice had significantly heavier visceral adipose tissue weight compared with lean mice (*P* < 0.0001). There were no significant differences in liver and kidney weight between the two groups. After generating diet-induced obese mice with greater body weight and heavier visceral adipose tissue, we conducted electrophysiological experiments using these mice. In the following electrophysiological experiments, the average body weight of lean mice was 39.5 ± 0.3 g (*n* = 52) and obese mice was 48.0 ± 0.9 g (*n* = 26); there was a significant difference between the two groups (*P* < 0.0001).

**Table 1:** Visceral organ weight of lean and obese mice.

	Lean mice (*n* = 14)	Obese mice (*n* = 9)	*P-*value
Liver (g)	2.09 ± 0.06	1.87 ± 0.11	N.S.
Kidneys (g)	0.65 ± 0.02	0.66 ± 0.02	N.S.
Total adipose tissue (g)	1.87 ± 0.09	3.78 ± 0.45	<0.0001
Epididymal	0.78 ± 0.05	1.92 ± 0.26	<0.0001
Perinephric	0.36 ± 0.03	0.83 ± 0.12	<0.001
Mesenteric	0.73 ± 0.04	1.03 ± 0.10	<0.01

Visceral organs were taken from 9–10 week-old mice. Values are ± SEM. N.S., not significantly different.

### Electrophysiological characteristics of VTA neurons in lean and obese mice

**Figure 2:**
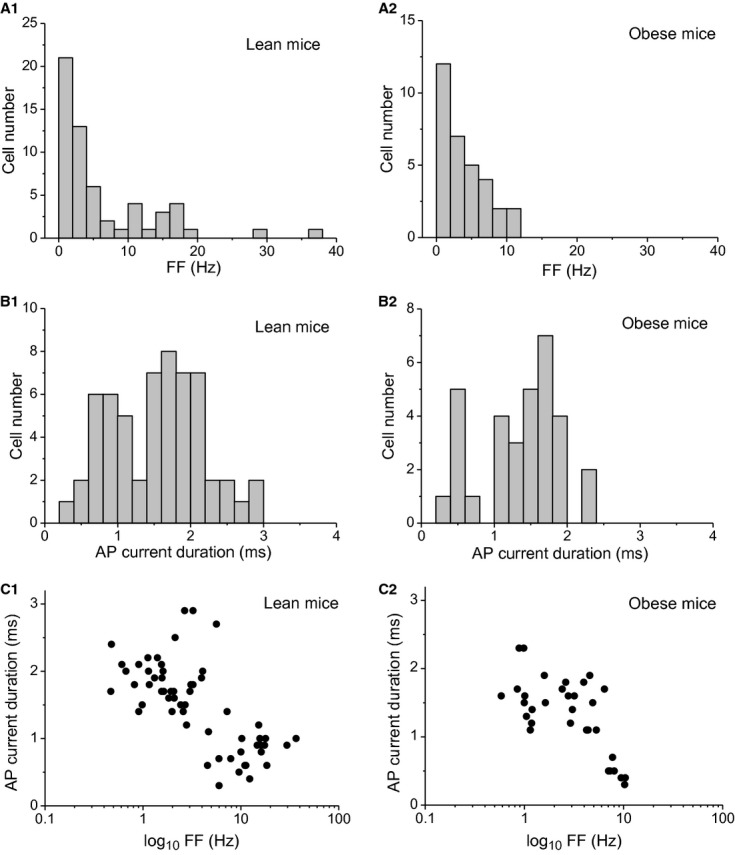
Electrophysiological characteristics of ventral tegmental area (VTA) neurons in lean and obese mice. (A_1_) Firing frequency (FF) histogram of VTA neurons in lean mice. (A_2_) FF histogram of VTA neurons in obese mice. Bin width is 2 Hz. (B_1_) Action potential (AP) current duration histogram of VTA neurons in lean mice. (B_2_) AP current duration histogram of VTA neurons in obese mice. Bin width is 0.2 msec. (C_1_) Relationship between FF and AP current duration of VTA neurons in lean mice. (C_2_) Relationship between FF and AP current duration of VTA neurons in obese mice. *x*-axes are presented in logarithmic scale.

Figure 2 shows electrophysiological characteristics of all VTA neurons in lean (*n* = 58) and obese mice (*n* = 32). The FF distribution exhibited bimodality in lean mice (Fig. 2A_1_), whereas a bimodal distribution of FF was not obvious in obese mice (Fig. 2A_2_). AP current duration of VTA neurons was distributed in a bimodal manner in lean (Fig. 2B_1_) and obese mice (Fig. 2B_2_). In lean mice, FF was negatively correlated with AP current duration (ρ = −0.702, *P* < 0.0001). After plotting the data, analysis showed that the distribution of AP current duration was divided into two groups at a cut-off value of 1.2 msec (Fig. 2C_1_). Similarly, FF distribution was divided into two groups at a cut-off value of 5 Hz (Fig. 2C_1_). Using FF and AP current duration cut-off values simultaneously, VTA neurons of lean mice were classified as group-1 neurons when they had FF <5.0 Hz and AP current duration > 1.2 msec (*n* = 34), and classified as group-2 neurons when they had FF ≧5.0 Hz and AP current duration ≦1.2 msec (*n* = 19). As FF was negatively correlated with AP current duration in obese mice (ρ = −0.631, *P* < 0.0001), the same cut-off values of FF and AP current duration were applied to VTA neurons of these mice: 16 group-1 neurons and eight group-2 neurons. Using the criteria, five VTA neurons in lean mice and eight VTA neurons in obese mice were unclassified.

### Firing properties of two groups of VTA neurons in lean and obese mice

**Figure 3:**
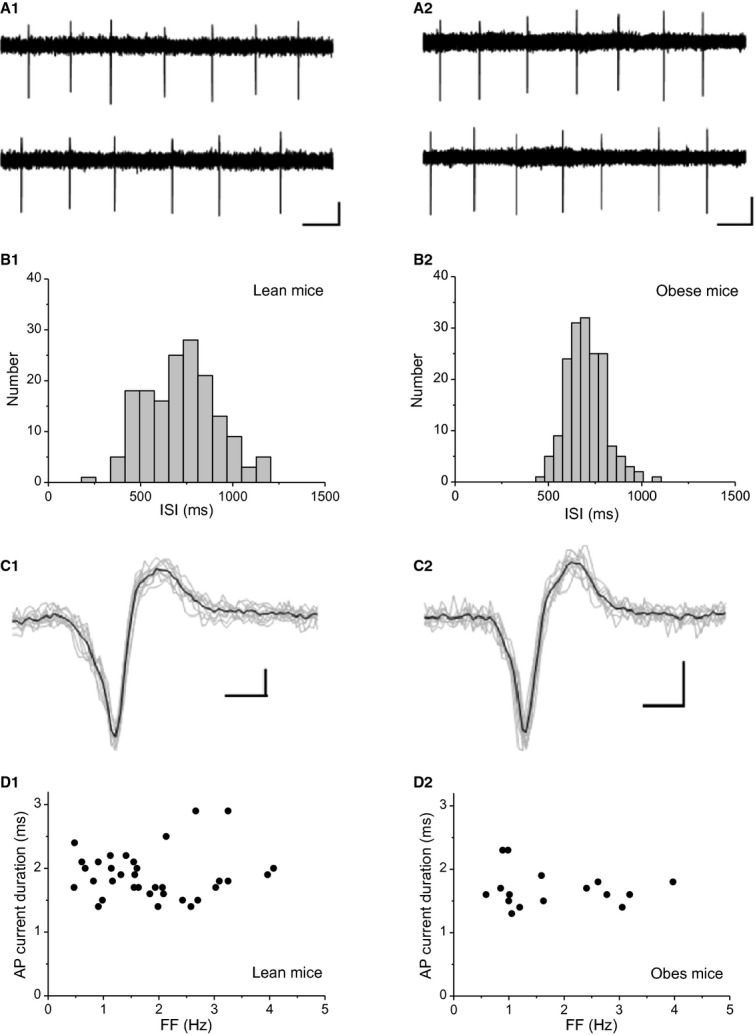
Spontaneous firing of VTA group-1 neurons in lean and obese mice. (A_1_) Spontaneous firing in lean mice. Scale bars, 20 pA; 0.5 sec. (A_2_) Spontaneous firing in obese mice. Scale bars, 20 pA; 0.5 sec. (B_1_) Interspike interval (ISI) histogram of lean mice. Bin width is 79 msec. (B_2_) ISI histogram of obese mice. Bin width is 48 msec. (C_1_) Average action potential (AP) current (*n* = 10) in lean mice. Scale bars, 10 pA; 1 msec. (C_2_) Average AP current (*n* = 10) in obese mice. Scale bars, 10 pA; 1 msec. (D_1_) Relationship between firing frequency (FF) and AP current duration in lean mice (*n* = 34). (D_2_) Relationship between FF and AP current duration in obese mice (*n* = 16).

Figure 3 shows the firing properties of VTA group-1 neurons in lean and obese mice. In lean mice, the VTA group-1 neurons spontaneously fired at 1.5 Hz (Fig. 3A_1_). A histogram of FF showed a peak at 750 msec (Fig. 3B_1_) and the AP current duration was 2.1 msec in lean mice (Fig. 3C_1_). In obese mice, VTA group-1 neurons spontaneously fired at 1.6 Hz (Fig. 3A_2_). A histogram of FF showed a peak at 700 msec (Fig. 3B_2_) and the AP current duration was 1.9 msec in obese mice (Fig. 3C_2_). Figure 3D shows a plot of FF and AP current duration of VTA group-1 neurons in lean (Fig. 3D_1_) and obese mice (Fig. 3D_2_).

**Figure 4:**
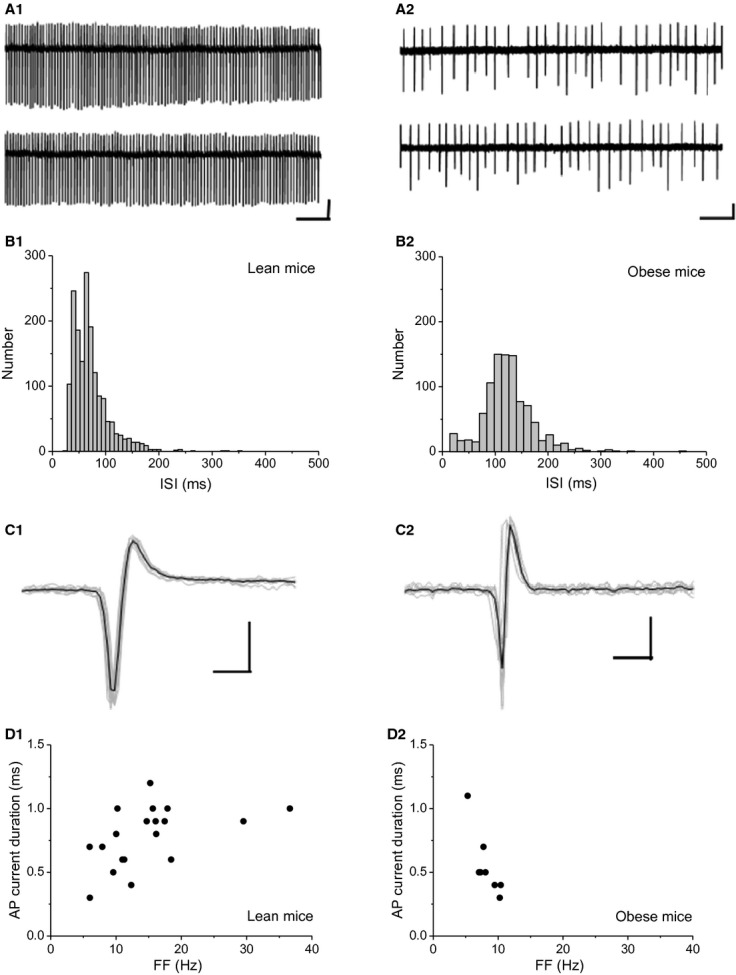
Spontaneous firing of ventral tegmental area (VTA) group-2 neurons in lean and obese mice. (A_1_) Spontaneous firing in lean mice. Scale bars, 100 pA; 0.5 sec. (A_2_) Spontaneous firing in obese mice. Scale bars, 40 pA; 0.5 sec. (B_1_) Interspike interval (ISI) histogram of lean mice. Bin width is 8 msec. (B_2_) ISI histogram of obese mice. Bin width is 14 msec. (C_1_) Average action potential (AP) current (*n* = 10) in lean mice. Scale bars, 100 pA; 1 msec. (C_2_) Average AP current (*n* = 10) in obese mice. Scale bars, 40 pA; 1 msec. (D_1_) Relationship between firing frequency (FF) and AP current duration in lean mice (*n* = 19). (D_2_) Relationship between FF and AP current duration in obese mice (*n* = 8).

Figure 4 shows firing properties of VTA group-2 neurons in lean and obese mice. In lean mice, VTA group-2 neurons spontaneously fired at 14.7 Hz (Fig. 4A_1_). A histogram of FF showed a peak at 60 msec (Fig. 4B_1_) and AP current duration was 0.9 msec in lean mice (Fig. 4C_1_). In obese mice, VTA group-2 neurons spontaneously fired at 8.1 Hz (Fig. 4A_2_). A histogram of FF showed a peak at 120 msec (Fig. 4B_2_) and AP current duration was 0.5 msec in obese mice (Fig. 4C_2_). Figure 4D shows a plot of FF and AP current duration of VTA group-2 neurons in lean (Fig. 4D_1_) and obese mice (Fig. 4D_2_).

Finally, we summarized the firing properties of VTA group-1 and group-2 neurons in lean and obese mice (Table 2). Obese and lean mice did not show any significant differences in firing properties of VTA group-1 neurons. In contrast, in VTA group-2 neurons, obese mice exhibited a lower FF (*P* < 0.005) (*F-*test, *P* < 0.001) and shorter AP current duration (*P* < 0.05) compared with lean mice.

**Table 2:** Firing properties of VTA neurons in lean and obese mice.

	Lean mice	Obese mice	*P-*value
Group-1 neurons	(n = 34)	(n = 16)	
FF (Hz)	1.9 ± 0.2	1.8 ± 0.3	N.S.
CV of ISI	0.17 ± 0.03	0.15 ± 0.03	N.S.
AP current duration (msec)	1.9 ± 0.1	1.7 ± 0.1	N.S.
Group-2 neurons	(n = 19)	(n = 8)	
FF (Hz)	14.8 ± 1.7	8.2 ± 0.6	<0.005
CV of ISI	0.25 ± 0.04	0.34 ± 0.05	N.S.
AP current duration (msec)	0.8 ± 0.1	0.6 ± 0.1	<0.05

Values are given as mean ± SEM. VTA, ventral tegmental area; FF, firing frequency; CV, coefficient of variation; ISI, interspike interval; AP, action potential; N.S., not significantly different.

## Discussion

In this study, we found that excitability of VTA group-1 neurons, which had both low FF and broad AP current duration, did not significantly change in diet-induced obese mice, while excitability of VTA group-2 neurons, which had both fast FF and short AP current duration, decreased in obese mice.

In this study, we found that VTA neurons had a negative correlation between FF and AP current duration in lean and obese mice. Using plots of the data, we successfully classified VTA neurons into two types using cut-off values of FF (5 Hz) and AP current duration (1.2 msec). Our cut-off value for AP current duration of VTA neurons is consistent with those used for classifying VTA dopamine and GABA neurons in previous studies by in vitro extracellular recordings (Ford et al. 2006; Chieng et al. 2011). The reported cut-off values are 0.8–0.9 msec (Chieng et al. 2011) and 1.2 msec (Ford et al. 2006). Using whole-cell recording from brain slice preparations, Margolis et al. (2012) showed that AP duration widely overlaps between VTA dopamine and GABA neurons. However, AP duration of these neurons distributes over 1 msec and no information is available on AP duration less than 1 msec. Although extracellular and whole-cell recordings of AP duration are highly correlated (Chieng et al. 2011; Margolis et al. 2012), overlap of AP duration between VTA dopamine and GABA neurons is more prominent in whole-cell recordings (Chieng et al. 2011). Our findings concerning AP current duration of VTA neurons are consistent with previous studies using in vivo extracellular recordings (Ungless et al. 2004; Luo et al. 2011). In anesthetized rats, Ungless et al. (2004) report a cut-off value of 1.1 msec for the classification of VTA dopamine and GABA neurons. Luo et al. (2011) reported broad and short AP duration in VTA dopamine and non-dopamine neurons, respectively, in anesthetized rats. In contrast, using extracellular recording in freely moving mice, Cohen et al. (2012) reported that AP duration overlapped between VTA dopamine and GABA neurons. It is likely that the AP duration of VTA neurons is affected by different recording methods and anesthesia. In extracellular recordings from brain slice preparations, AP duration is considered a useful primary criterion to classify putative VTA dopamine and GABA neurons. Our FF cut-off value for classification was not wholly consistent with those for classifying VTA dopamine and GABA neurons in previous studies (Ungless et al. 2004; Ford et al. 2006; Chieng et al. 2011; Margolis et al. 2012). The FF of VTA GABA neurons has been reported to be 3.6 Hz (Chieng et al. 2011), more than 10 Hz (Ford et al. 2006), 2.6 Hz (Ungless et al. 2004), and 3.3 Hz (Margolis et al. 2012). Previous studies also show an overlap of FF between VTA dopamine and GABA neurons (Ungless et al. 2004; Chieng et al. 2011; Margolis et al. 2012). In this study, we did not count silent VTA neurons. It has been reported that a considerable population of VTA GABA neurons are not spontaneously active; the population rates of the silent neurons have been variously reported as 22% (Chieng et al. 2011) and 65% (Margolis et al. 2012). Therefore, when focusing on spontaneously active VTA GABA neurons, differences in the FF results may become smaller. In the study by Chieng et al. (2011), the majority of FFs of VTA GABA neurons distributed from 5 to 15 Hz where silent neurons will be excluded, while the FF of VTA non-GABA neurons is <5 Hz. FF can provide useful secondary information when identifying putative VTA dopamine and GABA neurons. In this study, it is notable that VTA neurons were not distinctively classified into two groups. This study has a potential limitation in that we did not conduct neurochemical identification of VTA neurons that were electrophysiologically recorded. Nevertheless, by examining FF and AP current duration simultaneously, we classified VTA neurons into two types and suggest that VTA group-1 and group-2 neurons are putative VTA dopamine and GABA neurons, respectively.

In this study, FF, firing regularity, and AP current duration of VTA group-1 neurons were not significantly altered in obese mice. Previous studies suggest that obese rodents, chronically fed with energy-rich diets, show decreased biochemical activity of VTA dopamine neurons, and subsequently reduced dopamine transmission in the NAcb (Geiger et al. 2009; Li et al. 2009). The differences in results likely depend on different dopamine effects between the NAcb and VTA. Synaptic dopamine release from nerve terminals in the NAcb is accelerated with APs conducted by axons of VTA dopamine neurons. Conversely, somatodendritic dopamine release via membrane depolarization activates dopamine (D2) autoreceptors to inhibit excitability of VTA dopamine neurons, thus exhibiting autoinhibition in the VTA (Mercuri et al. 1997; Adell and Artigas 2004). In obesity, decreased TH in VTA dopamine neurons is responsible for a reduction in the release of dopamine from nerve terminals in the NAcb (Geiger et al. 2009; Li et al. 2009), whereas decreased dopamine biosynthesis in somatodendritic regions of VTA dopamine neurons attenuates D2 receptor-mediated autoinhibition, and recovers excitability of these neurons. In this study, the FF and AP current duration of VTA group-2 neurons significantly decreased in obese mice, while firing regularity was similar between the two groups. In obese mice, the homogeneity of variance for FF of VTA group-2 neurons was significantly greater compared with lean mice FF of VTA group-2 neurons, which had higher FFs (over 10 Hz), was selectively decreased. Thus, diet-induced obesity may selectively affect a subpopulation of putative VTA GABA neurons. However, an immunohistochemical study showed that VTA GABA neurons are divided into several neuronal subpopulations in accordance with Ca^2+^-binding protein contents (Olson and Nestler 2007). There are other populations of VTA GABA neurons in addition to those forming a local neuronal circuit in the VTA or projecting to the NAcb. For example, GABA projections to the prefrontal cortex (Carr and Sesack 2000), dorsal raphe nucleus, and the periaqueductal gray (Kirouac et al. 2004) have been reported. Moreover, there is no clear evidence that the neurons that make local connections in the VTA are true interneurons, or whether some populations of projection neurons leave local collaterals; further studies are needed. Our findings concerning VTA group-2 neurons in obese mice show that diet-induced obesity is likely to affect ion channel properties, which are significant for and specific to maintaining the FF and AP current duration. An electrophysiological study on substantia nigra (SN) GABA neurons showed that tetrodotoxin-sensitive Na^+^ channels regulate FF and ω-conotoxin-sensitive Ca^2+^ channels, which couple with apamin-sensitive Ca^2+^-dependent small conductance K^+^ channels, and regulate both FF and firing regularity (Atherton and Bevan 2005). Decreased Na^+^ channel activity may contribute to a reduction in the excitation of putative VTA GABA neurons in diet-induced obese mice. Khaliq and Bean (2010) found that SN and VTA dopamine neurons have different channels that control pacemaking. As in the case of VTA dopamine neurons, it is notable that spontaneous activity in VTA GABA neurons is not necessarily driven by the same channels present in SN GABA neurons.

Recent studies have shown that VTA GABA neurons are important for the regulation of reward-related behavior (Brown et al. 2012; Van Zessen et al. 2012). Brown et al. (2012) showed that activation of VTA GABA neurons mediates aversive behavior. Van Zessen et al. (2012) showed that activation of VTA GABA neurons terminates the reward consumption of sucrose. Obesity-induced decreases in the excitability of VTA group-2 neurons, presumed to be GABAergic in this study, may contribute to reward-related behavior. In conclusion, in this study we have shown that diet-induced obesity decreased excitability of putative VTA GABA neurons. This altered GABA function in the VTA may contribute to the pathophysiology of reward-related feeding in obesity.
